# The effect of HMGB1 on the clinicopathological and prognostic features of cervical cancer

**DOI:** 10.1042/BSR20181016

**Published:** 2019-05-02

**Authors:** Pan Li, Mengfei Xu, Hongbing Cai, Niresh Thapa, Can He, Ziye Song

**Affiliations:** Department of Gynecological Oncology, Zhongnan Hospital of Wuhan University, Hubei Cancer Clinical Study Center, Hubei Key Laboratory of Tumor Biological Behaviors, P.R. China

**Keywords:** bioinformatics analysis, cervical cancer, HMGB1, prognosis

## Abstract

Cervical cancer is the third leading cause of cancer death among women in less-developed regions. Because of the poor survivorship of patients with advanced disease, finding new biomarkers for prognostic prediction is of great importance. In the current study, mRNA datasets (GSE9750 and GSE63514) were retrieved from Gene Expression Omnibus and was used to identify differentially expressed genes. The underlying molecular mechanisms associated with high-mobility group box 1 protein (HMGB1) were investigated using bioinformatics analysis. Immunohistochemical analysis of HMGB1 was performed on 239 cases of cervical cancer samples to investigate its possible correlation with clinicopathological characteristics and outcomes. A preliminary validation has been made to explore the possible correlation factors with HMGB1 that promote migration of cervical cancer cells. Bioinformatics analysis showed that adherens junction was significant for both *P*-value and enrichment scores, which was consistent with the clinical study. The underlying molecular mechanisms might be the interaction among HMGB1, RAC1, and CDC42. HMGB1 expression was significantly associated with tumor size, parametrial infiltration, the depth of cervical stromal invasion, and FIGO stage (*P*=0.003, 0.019, 0.013, and 0.003, respectively). FIGO stage, lymph mode metastasis, and HMGB1 expression were independent predictors of a poorer prognosis of patients with cervical cancer. Knockdown of HMGB1 inhibits migration of Siha and C33A cells *in vitro*. Western blot and quantitative real-time PCR (qRT-PCR) showed that the expression of RAC1 and CDC42 was positively correlated with HMGB1. HMGB1 is a useful prognostic indicator and a potential biomarker of cervical cancer. RAC1 and CDC42 may be involved in the progression of cervical cancer migration induced by HMGB1.

## Introduction

Cervical cancer is the fourth most common diagnosed female malignancy, and is the third leading cause of cancer death among women in less-developed regions [1]. The natural history of cervical cancer has been deeply investigated; the persistent infection of high-risk types of human papillomavirus (HPV) plays an important role in the oncogenesis. The suitable screening tests of cervical cancer – HPV detection and liquid-based cytology (LBC), and biopsy – make it possible to detect the pre-cancerous lesions and intervene in an early stage [2]; however, cervical cancer remains a considerable problem that threaten the health of women. Although the early-stage cancer can be cured by radical hysterectomy, chemotherapy, and radiotherapy [3]; patients with advanced disease still have limited choice and poor survivorship [4]. Several pathways have been found involved in the progress of cervical cancer, but no biomarker has been proved to be significantly associated with the clinical outcome of cervical cancer. Therefore, identifying new prognostic factors, like molecular biomarkers, would be helpful to pre-therapeutically stratify patients for specific-targeted therapies.

Because of the development of sequencing technology and the limitation of the experiment, bioinformatics shows a promising potential in cancer research. By analyzing the association between clinical data and molecular mechanism; new biomarkers for early diagnosis, therapeutic evaluation, and for prognosis can be found.

The high-mobility group box 1 protein (HMGB1) belongs to the group of chromatin-associated proteins with high acidic and basic amino acid content [5], which is present in the nucleus and cytoplasm of nearly all types of cells. Intracellular HMGB1 is a key participant in fundamental nuclear events such as DNA recombination, replication, remodeling, and repairing [6–8]. Extracellular HMGB1 can activate polymorphonuclear leukocytes (PMN), mononuclear macrophages and natural killer (NK) cells [9], promote the release of inflammatory mediators, also induce maturation and migration of immature dendritic cells (DC) and the polarization of T lymphocytes [10–14]. Several clinical studies have proved that HMGB1 is overexpressed in a variety of human neoplasms, including breast, colorectal, hepatocellular/biliary, lung, and pancreatic cancer [15]. In most cases, extracellular HMGB1 binds to various receptors such as Toll-like receptors (TLRs) and receptor for advanced glycation end products (RAGE), which appears to be important in cancer progression [16,17], especially in proliferation and migration of tumor cells, and in the process of angiogenesis and regeneration [18]. Recently, several studies demonstrated that, within the cytosol, HMGB1 also promoted autophagy (a conserved programmed survival pathway evoked following environmental and intracellular stress), which in tumor cells blocks the apoptosis signal of the mitochondria and contributes to the resistance to radiotherapy and chemotherapy [19–21].

The present study is to investigate the prognostic value of HMGB1 expression in cervical cancer and to further understand its role in the development of cervical cancer.

## Materials and methods

### Ethical statement for human cervical cancer tissues

The experiments using human cervical cancer and paracancerous tissues for RNA isolation and quantitative real-time PCR (qRT-PCR) was approved by the Ethic Committee at Zhongnan Hospital of Wuhan University.

### Study design and data collection

The expression profiles of mRNA of cervical cancer were downloaded from Gene Expression Omnibus (GEO, http://www.ncbi.nlm.nih.gov/geo/). The dataset GSE9750 (*n*=33) and GSE63514 (*n*=68) were obtained from the same microarray platform (Affymetrix Human Genome U133A Array). Raw expression data were download from GEO. Samples of the two microarray was divided into two groups according to the level of HMGB1 expression the cut-off criteria was defined as quartile: HMGB1 high-expression group and HMGB1 low-expression group.

### Identification of differentially expressed genes (DEGs)

The GEO online tool GEOR2 was used to find the differentially expressed genes (DEGs) between the two groups. The significance analysis of microarrays (SAM) with false discovery rate (FDR) < 0.05 and |log2 fold change (FC)| > 1 were applied to selected genes.

### Functional and pathway enrichment analysis

For a more in-depth investigation of the functions of DEGs, the Database for Annotation, Visualization and Integrated Discovery (DAVID) database (http://david.abcc.ncifcrf.gov/) was used to enrich biological themes on GO terms and on KEGG pathway maps. *P*<0.05 was set as the cut-off criterion.

### Protein–protein interaction (PPI) network of DEGs

In order to find the hub genes within the gene regulatory networks, protein–protein interaction (PPI) network of DEGs was constructed by Search Tool for the Retrieval if Interacting Genes (STRING) Database (http://string-db.org/) and subsequently was visualized by Cytoscape software. The interactions between HMGB1 and other key genes were drawn by cBioPortal (http://www.cbioportal.org/index.do).

### Preparation of the human cervical cancer samples

The cervical cancer tissues samples were collected from patients undergoing radical hysterectomy at Zhongnan Hospital of Wuhan University. Two pathologists histologically diagnosed the tissues independently. The cervical cancer tissues were stored in liquid nitrogen or fixed with 4% PFA. Written informed consents were obtained from all subjects. The Ethic Committee at Zhongnan Hospital of Wuhan University approved the study using cervical cancer samples.

### Immunohistochemical (IHC) analysis of HMGB1

Immunohistochemical (IHC) staining procedure was performed as previously described [22]. Tissue sections were incubated overnight in rabbit anti-HMGB1 polyclonal antibody (1:300, Abcam, Cambridge, U.K.) at 4°C and incubated with an anti-rabbit secondary antibody (1:1000, Abcam, Cambridge, U.K.) for 30 min at 37°C on the following day. Based on the staining intensity and the proportion of positive cells, IHC scores for HMGB1 were measured by two pathologists with no prior knowledge of clinicopathological results, and any discrepancies in scores were discussed until a consensus was reached. Five visual field (10*40) were chosen randomly and every case was scored by combining intensity points and proportion points. Proportion of positive cells were classified into four categories: 1 point (≤25%), 2 points (26–50%), 3 points (51–75%), and 4 points (>75%). Then the intensity of nuclear or cytoplasmic staining were scored as follows: weak staining detectable above background scored 1, moderate staining scored 2, and strong staining scored 3. We took the mean scores, which combined intensity points with proportion points of five visual fields as the final scores. Cases with final scores over 4 were regarded as high expressions of HMGB1

### Cell culture and sh-RNA transfection

Cervical cancer cell line Siha and C33A were maintained in Hubei Cancer Clinical Study Center, China. Cells were cultured in DMEM (Hyclone, U.S.A.) supplemented with 10% fetal bovine serum (Gibco, U.S.A.) at 37°C with 5% CO_2_. The human HMGB1 shRNA plasmid and non-silencing negative control (NC) shRNA plasmid were obtained from GenePharma Co. (Shanghai, China). The relevant shRNA template oligonucleotides sequences are shown in Table 3. For temporal transfection, cells (60–70% confluence) were transfected with 2 μg of plasmid using Lipofectamine 2000 (Invitrogen, U.S.A.) according to the manufacture’s instructions. Cells were harvested in 48 h.

### Migration assays and wound healing

Cell migration assays, which were performed using a Transwell permeable supports system and wound healing assays, referred to the procedures in the study of Zhao et al. [23]. The number of migratory cells and wound closure were measured by Image J.

### Quantitative real-time PCR (qRT-PCR)

RNAs were isolated from Siha and C33A cell lines in different groups. Reverse transcription-PCR (RT-PCR) was performed using the RT-PCR kit (TaKaRa, Japan) for the detection of HMGB1, RAC1, and CDC42 transcript levels according to the instructions, and glyceraldehyde-3-phosphate dehydrogenase (GAPDH) as a control housekeeping gene. Forward and reverse primers were added at a concentration of 0.2 pmol/ml per 25 μl. HMGB1: forward: 5′-GGAGAGATGTGGAATA-3′ reverse: 5′-GGGAGTGAGTTGTGTA-3′; RAC1: forward: 5′-GCGGCACCACTGTCCCAACA-3′ reverse: 5′-AGCGCCGAGCACTCCAGGTA-3′; forward: 5′-GGCGATGCTGTTGGTAA-3′, reverse: 5′-GCGGTCGTAATCTGTCATAATCCT-3′.

### Western blot

Immunoblotting was done as standard procedures. Cell lysates was separated using SDS-gel electrophoresis and transferred onto PVDF membranes. They were blocked with 5% BSA and incubated with specific antibodies. The following primary antibodies were used: HMGB1 (1:1000; ab5662; Abcam, Cambridge, U.K.), rac1 (1:1000; ab155938; Abcam, Cambridge, U.K.), and cdc42 (1:1000; ab64533; Abcam, Cambridge, U.K.). All Western blots shown were representative results obtained from at least three independent experiments, and all results were analyzed by Image J.

### Statistical analysis

Pearson’s chi-square test or Fisher’s exact test were used to examine the correlations between HMGB1 expression and clinicopathological characteristics. Overall survival (OS) and disease free survival (DFS), which were measured from the date of surgery, were established using the Kaplan–Meier method and were compared by the log-rank test. The multivariate analysis was conducted through Cox proportional hazards model. Cinicopathologic factors, which played significant statistical roles in univariated analysis, were comprised as co-variables in multivariate analysis. Hazard ratios (HRs) and 95% confidence intervals (CIs) were evaluated for each factor. All of the above tests were performed using the Statistical Package for the Social Sciences (SPSS) Version 20.0 (IBM Corp., U.S.A.), and *P*<0.05 was considered as significant.

## Results

### Identification of DEGs in cervical cancer tissues

**Figure 1:**
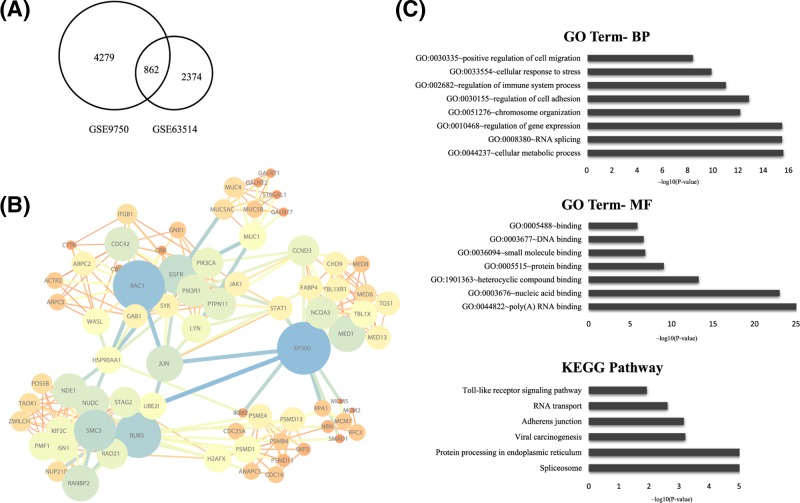
Bioinformatics analysis of DEGs (**A**) Flowchart of bioinformatics analysis. 862 integrated DEGs between two groups were chosen as the final DEGs. (**B**) PPIs network of DEGs. DEGs in the PPI networks with a connectivity degree >20 and with the combination score >0.9 were represented, of which the top 8 high-interaction score genes were identified as real hub genes. (**C**) Go analysis of DEGs showed the significantly over-represented biological processes, molecular function of DEGs, and KEGG pathway enrichment of DEGs showed the significantly over-represented pathways in DEGs.

The gene expression profile of GSE9750 including 33 primary tumor tissues, and the gene expression profile of GSE63514 including 40 CIN3 lesions and 28 cancers specimens tissues were analyzed. Using the GEO online tool GEOR2, selecting *P*<0.01 and |logFC| > 1 as the cut-off criteria, integrated DEGs between two profiles were chosen as the final DEGs. A total of 862 DEGs that exhibited the same expression trends in two profiles were identified in the high HMGB1 expression group in cervical cancer (Figure 1A).

### PPI network construction

We constructed a network of PPI of DEGs by STRING and then validated using Cytoscape. Of around 1030 genes, DEGs in the PPI networks with a connectivity degree >20 and with the combination score >0.9 were represented. Overall, we identified top high-interaction score genes as hub genes, which were CDC42, RAC1, EGFR, JUN, EP300, BUB3, SMC3, and MED1 (Figure 1B).

### Functional enrichment analysis

To have a more in-depth understanding of the DEGs of cervical cancer, the up- and down-regulated DEGs were respectively uploaded into the DAVID database. GO analysis showed that the genes were significantly enriched for *P*-value in biological processes in positive regulation of cell migration, cellular response to stress, regulation of immune system process, regulation of cell adhesion, chromosome organization, regulation of gene expression, RNA splicing, and cellular metabolic process. As for the molecular function, genes were significantly enriched in DNA binding, small molecule binding, protein binding and poly(A) RNA binding. These biological processes and molecular functions had the highest enrichment scores that involved in the negative regulation of interleukin-2 secretion, CRD-mediated mRNA stabilization and spindle localization. KEGG pathway analysis showed that TLR signaling pathway, RNA transport, adhesion junction, viral carcinogenesis, protein processing in endoplasmic reticulum, and spliceosome were most significantly enriched. The expression level of HMGB1 is related to invasion and migration (Figure 1C).

### Patients’ characteristics

A total of 239 patients with cervical cancer underwent radical hysterectomy at Zhongnan Hospital of Wuhan University from 2006 to 2015 were enrolled. No patient received neoadjuvant chemotherapy or neoajuvant radiotherapy. General characteristics of patients in the present study were showed in Table 1. The median age was 44.4 ± 12.4 years with maximum 68 years and minimum 21 years, 65.69% (156/239) were over 40 years. Histopathologically, 86.61% (207/239) of cases were of cervical squamous cell carcinoma, and 13.39% (32/239) were of adenocarcinoma. The clinical staging was carried out according to the FIGO staging for cervical cancer: 151 cases (63.18%) were stage I and 88 cases (36.82%) were stage II. The pathological grade was classified into groups according to the WHO classification, with 67 (28.03%) poorly-differentiated, 111 (46.44%) moderately differentiated, and 61 (25.52%) highly differentiated carcinoma. The median follow-up period was 56 months (ranging from 12 to 120 months). During follow-up, patients who died of other diseases and accidents were excluded.

**Table 1:** Associations between tissue HMGB1 expression and clinicopathologic characteristics of cervical cancer patients

Variables	HMGB1 expression (n (%))		Total N	χ^2^	*P*-value
	High 5–7	Low 0–4			
**Age at diagnosis (years)**				0.262	0.609
** <40**	47 (57.3)	35 (42.7)	82		
** ≥40**	84 (53.5)	73 (46.5)	157		
**Tumor size**				8.627	0.003
** <4cm**	60 (46.2)	70 (53.8)	130		
** ≥4cm**	71 (65.1)	38 (34.9)	109		
**Vascular invasion**				0.291	0.589
** Yes**	53 (57.0)	40 (43.0)	93		
** No**	78 (53.4)	68 (46.7)	146		
**Parametrial infiltration**				5.459	0.019
** Yes**	17 (73.9)	6 (26.1)	23		
** No**	11 (52.8)	102 (47.2)	216		
**Histological subtype**				0.311	0.577
** Squamous cell carcinoma**	112 (54.1)	95 (45.9)	207		
** Adenocarcinoma**	19 (59.4)	13 (40.6)	32		
**Histopathologic grade**				2.463	0.292
** G1**	31 (46.3)	36 (53.7)	67		
** G2**	43 (38.7)	68 (61.3)	111		
** G3**	20 (32.8)	41 (67.2)	61		
**Depth of cervical stromal invasion**				6.130	0.013
** <1/2**	59 (47.2)	66 (53.8)	125		
** ≥1/2**	72 (63.2)	42 (36.8)	114		
**FIGO stage**				8.988	0.003
** I**	71 (47.0)	80 (53.0)	151		
** II**	60 (67.0)	28 (37.0)	88		
**Lymph node metastasis**				0.258	0.612
** Yes**	20 (58.8)	14 (41.2)	34		
** No**	111 (54.1)	94 (45.9)	205		

### Survival analysis

**Figure 2:**
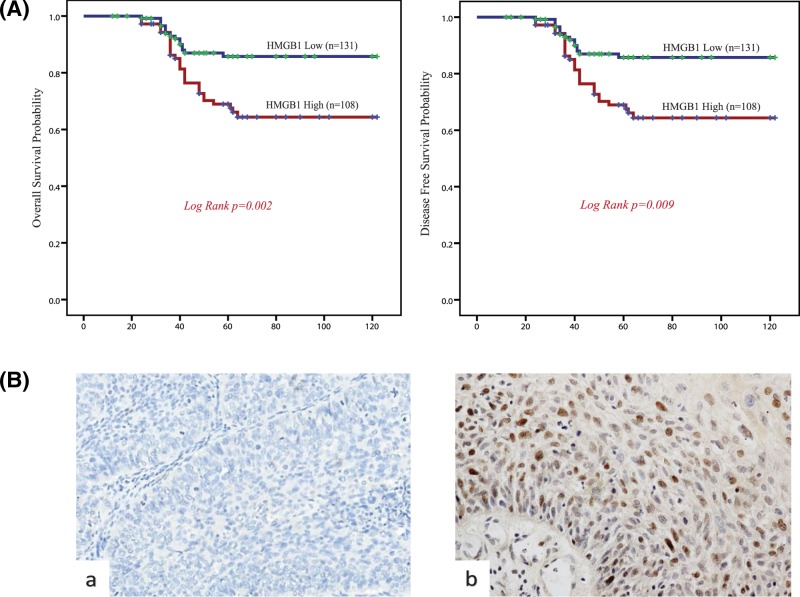
Clinical-pathological analysis (**A**) Survival curves using the Kaplan–Meier method by log-rank test for HMGB1 expression. OS rate was analyzed in 239 cervical cancer patients in relation to HMGB1 expression. Log-rank test *P-*value: 0.002. And disease-free time rate were analyzed in 239 cervical cancer patients in relation to HMGB1 expression. Log-rank test *P-*value: 0.009. (**B**) Representative immunohistochemistry samples of cervical cancer tissues demonstrating HMGB1 expression. Normal cervix tissue showed the negative HMGB1 staining reactivity. And cervical cancer tissue showed the positive HMGB1 staining reactivity. Magnification, ×400.

The Kaplan–Meier survival curves demonstrated that high HMGB1 expression group had a significantly lower OS and DFS rate compared with low HMGB1 expression group(*P*=0.002, *P*=0.009, respectively) (Figure 2A). Furthermore, Cox univariate proportional hazards analysis showed that higher level of vascular invasion, parametrial infiltration, the depth of cervical stromal invasion, FIGO stage, lymph node metastasis, and HMGB1 expression predicted significantly shorter OS and DFS. Importantly, multivariate analysis showed that FIGO stage, lymph mode metastasis, and HMGB1 expression were independent predictors of shorter OS and DFS (Table 2). These findings indicated that HMGB1 expression may be a useful marker for predicting the survival of patients with cervical cancer.

**Table 2:** The Cox proportional hazard regression analyses for OS and DFS of patients with cervical cancer

Variables	OS				DFS			
	Univariable analysis		Multivariable analysis		Univariable analysis		Multivariable analysis	
	HR (95% CI)	*P*-value	HR (95% CI)	P-value	HR (95% CI)	P-value	HR (95% CI)	P-value
Age at diagnosis	1.005 (0.984–1.027)	0.616			1.006 (0.989–1.023)	0.610		
Tumor size	1.438 (0.837–2.472)	0.188			1.223 (0.789–1.895)	0.209		
Vascular invasion	1.791 (1.044–3.072)	**0.034**	1.563 (0.869–2.812)	0.136	1.771 (1.152–2.722)	**0.031**	1.498 (0.960-2.338)	0.149
Parametrial infiltration	3.858 (1.925–7.734)	**0.003**	2.062 (0.937–4.541)	0.072	2.347 (1.240–4.443)	**0.000**	1.794 (0.902–3.568)	0.080
Histological subtype	1.080 (0.509–2.292)	0.841			1.658 (0.958–2.870)	0.863		
Histopathologic grade	1.068 (0.748–1.524)	0.718			0.973 (0.734–1.290)	0.773		
Cervical stromal invasion	2.248 (1.294–3.907)	**0.004**	1.382 (0.738-2.588)	0.312	2.125 (1.373–3.287)	**0.004**	1.654 (0.995–2.749)	0.052
FIGO stage	2.015 (1.173–3.463)	**0.011**	1.953 (1.031–3.699)	**0.040**	1.277 (0.822–1.986)	**0.013**	1.895 (1.003–3.579)	**0.049**
Lymph node metastasis	2.434 (1.468–4.036)	**0.001**	2.146 (1.139–4.044)	**0.018**	2.464 (1.488-4.082)	**0.000**	2.126 (1.131-3.997)	**0.019**
HMGB1 expression	0.634 (0.412–0.976)	**0.039**	0.354 (0.198–0.864)	**0.000**	0.631 (0.410–0.972)	**0.011**	0.544 (0.350–0.846)	**0.007**

*P-*value less than 0.05 indicates statistical significance.

**Table 3:** Target site and sequences of shRNA

pGPU6/GFP/Neo-HMGB1-homo-539	
Target sequence 1	GCGAAGAAACTGGGAGAGATG
Sense strand 5′-3′	GCGAAGAAACTGGGAGAGATGTTCAAGAGACATCTCTCCCAGTTTCTTCGCTTTTTTG
Antisense strand 3′-5′	CGCTTCTTTGACCCTCTCTACAAGTTCTCTGTAGAGAGGGTCAAAGAAGCGAAAAAAC
pGPU6/GFP/Neo-shNC	
Target sequence 1	GTTCTCCGAACGTGTCACGT
Sense strand 5′-3′	GTTCTCCGAACGTGTCACGTTTCAAGAGAACGTGACACGTTCGGAGAATTTTTTG
Antisense strand 3′-5′	CAAGAGGCTTGCACAGTGCAAAGTTCTCTTGCACTGTGCAAGCCTCTTAAAAAAC

### Clinicopathological features and HMGB expression

Overexpression of HMGB1 was detected in 131 samples (45.19%), while 108 samples (54.81%) with low immunoreactivity. The positive particles were mainly located in the nucleus, with weak staining in the cytoplasm (Figure 2B). The association between clinicopathological characteristics and HMGB1 expression (low vs high expression) was summarized in Table 1. HMGB1 expression was positively associated with tumor size, parametrial infiltration, the depth of cervical stromal invasion and FIGO stage (*P*=0.003, 0.019, 0.013, and 0.003, respectively). There was no significant association between HMGB1 expression and histological subtype and grade, lymph node metastasis, ages, vascular invasion, and histopathologic grade.

### Down-regulation of HMGB1 inhibits migration of Siha cells *in vitro*

**Figure 3:**
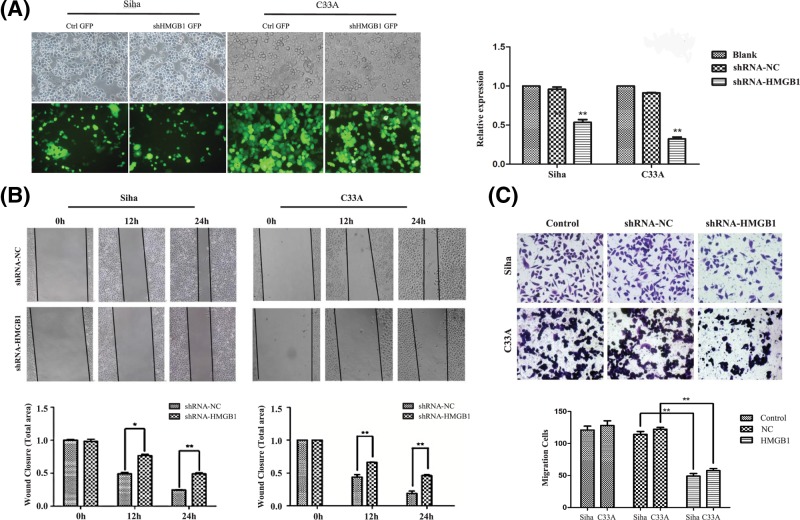
*In vitro* experiment of cervical cell lines (**A**) The transfection efficiency of shRNAs in Siha and C33A cell lines (×200 magnification). The transfection efficiency of shRNA in Siha and c33a cell line. The level of HMGB1 expression was determined in cervical cancer cells upon transfection with shHMGB1 or NC scramble by qRT-PCR. (**B**–**D**) Wound-healing assay images of wounds of Siha/C33A cells and Siha/C33A cells transfected with the specific shRNA against HMGB1 taken 0, 12, and 24 h after the wounds were inflicted (analyzed by *t*tests) (×100). ^*^P<0.05,^**^P<0.01. (**C**) Transwell assay. The migration cells were fixed in 75% alcohol and stained with crystal violet. The representative fields were photographed and counted at ×200 magnification. The cells were counted in five different fields per assay under the microscope. The migration cells in shRNA HMGB1 group was significantly less than those of control group. ^**^P<0.01

Siha and C33A cells were treated with the shRNA which leads knockdown of HMGB1 and the efficiency of the interference was confirmed by qRT-PCR. The transfection efficiency of siRNAs in Siha cells was over 80% and the transfection efficiency of siRNA in C33A cells was over 95% (Figure 3A,B). The specific shRNA was shown to induce a significant down-regulation in Siha and C33A cells (Figure 3A). After the HMGB1 knockdown, a wound healing assay showed that knockdown of HMGB1 in Siha and C33A cells resulted in cells migrating less frequently and could not grow to confluency after 48 h (Figure 3B). Furthermore, a Transwell assay also showed that the migration of Siha and C33A cells were significantly reduced compared with the control group (Figure 3C). Based on these assays, HMGB1 is associated with cell migration of cervical cancer cells.

### HMGB1 facilitates the expression of RAC1 and CDC42

**Figure 4:**
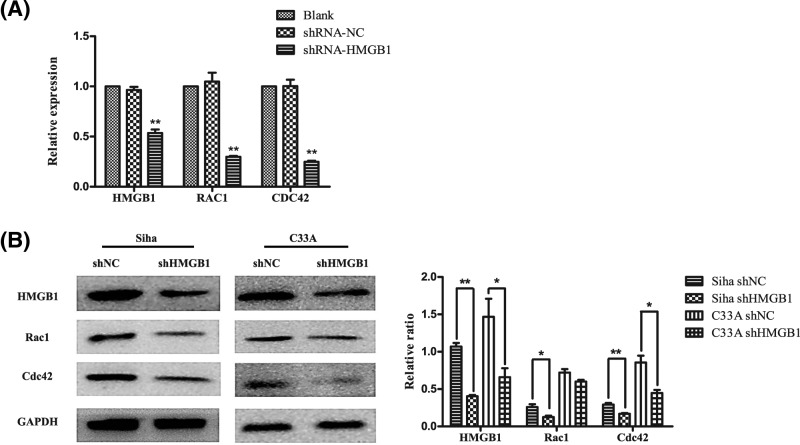
The expression of HMGB1 and relative genes (**A**) HMGB1, RAC1, and CDC42 protein and mRNA expression were detected in shRNA-NC and shRNA-HMGB1 in cervical cancer cell lines. Relative expression of HMGB1, RAC1, and CDC42 mRNA were detected by qRT-PCR. The data were normalized to the level of GAPDH mRNA. Error bars represent SD (*n*=3). (**B**) Immunoblot of HMGB1, Rac1, and Cdc42 in Siha/C33A cells after transfection with shRNA of HMGB1 compared with control group. Error bars represent SD (*n*=3). ^*^P<0.05, ^**^P<0.01.

PPI network in our study showed that CDC42 and RAC1, which are central to dynamic actin cytoskeletal assembly and rearrangement that are the basis of cell–cell adhesion and migration, were hub genes according to differentiate expression level of HMGB1. To further investigate the significance of HMGB1 in the expression of RAC1 and CDC42 in cervical cancers, RAC1 and CDC42 mRNA and protein were explored by qRT-PCR and Western blot analysis on HMGB1-silenced cells. After treatment with specific shRNA against HMGB1 for 48 hours, the relative expression of HMGB1 was significantly inhibited (Figure 4A). HMGB1 was significantly inhibited at mRNA and protein levels in transfected cells with HMGB1-shRNA compared with NC-shRNA group (*P*<0.01). At the same time, the mRNA and protein levels of RAC1 and CDC42 was lower in the HMGB1-shRNA transfected cells compared with NC-shRNA group (*P*<0.05, Figure 4B). Thus, the result revealed that HMGB1 facilitated the expression of RAC1 and CDC42.

## Discussion

The main factors that affect the prognosis of cervical cancer patients are tumor type, pathological grade, and clinical stage. However, it has been shown that other factors like molecular and cellular characteristics of primary tumor may improve the prognostic evaluation [24]. Tissue or serum HMGB1 has been proved to be highly expressed in a variety of cancers, including laryngeal squamous cell carcinoma, gastric cancer, pancreatic cancer, colorectal cancer, and cervical cancer [25]. Xu et al. [26] found that both nuclear and cytoplasmic HMGB1 were independent factors for poor prognosis in early-stage squamous cervical cancer. This conclusion is similar to ours that FIGO stages, lymph mode metastasis, and HMGB1 expression were independent predictors that lead to diverse OS and DFS among cervical cancer patients. The high HMGB1 level may play a role in the development of cervical cancer or even be a key point of oncogenesis, making it a potential target for cancer therapy.

In the present study, HMGB1 expression was significantly associated with tumor size, parametrial infiltration, the depth of cervical stromal invasion, and FIGO stage, while KEGG pathway analysis showed that cell cycle, spliceosome, DNA replication, adherens junction, Fc-γ R-mediated phagocytosis, and pancreatic cancer pathway possessed both significant *P*-values and higher enrichment scores. It reminds that the expression of HMGB1 may be associated with proliferation and metastasis of cervical cancer cells. Transwell chamber and wound-healing assay in our study were taken to reveal that high expression of HMGB1 promoted the migration of Siha cells. So we speculated that high expression of HMGB1 promoted invasion of tumor cells through signaling pathway related to cell adhesion and migration. Previous studies [27,28] showed that the mechanisms through which HMGB1 promotes the development of cervical cancer may include the following: HMGB1-activated p38, JNK, and mitogen-activated protein kinases (MAPKs), which further activated MMP-2 and MMP-9, promoting the degradation of extracellular matrix (ECM), tumor invasion, and metastasis by forming a complex with RAGE. HMGB1 binds with TLR, activating myeloid differentiation primary response gene 88 (MyD88), and finally activates NF-kB, which promotes the proliferation, invasion, and metastasis of tumor cells. Phosphatidylinositol 3-kinase/Akt (PI3K/AKT) pathway, which is promoting proliferation of tumor cells by regulating cell cycle 1 and is closely related to the invasion and metastasis of tumor, also reported to be mediated by HMGB1 [29,30].

Bioinformatics analysis in the present study also showed the possible mechanisms through which HMGB1-promoted tumor cell invasion and migration might interact with CDC42 and RAC1, which are central to dynamic actin cytoskeletal assembly and rearrangement that are the basis of cell–cell adhesion and migration. Then further investigated by RT-qPCR and Western blot on HMGB1-silenced cells revealed that the expression level of HMGB1 was positively correlated with RAC1 and CDC42, which suggested that there might be underlying mechanisms of interaction among HMGB1, RAC1, and CDC42 in promoting tumor cell migration. Taguchi et al. [31,32] confirmed that HMGB1 interacted with RAGE and then activated some intracellular transduction pathways, including the MAPKs family, cell-cycle proteins (Cdc42), Rac, and NF-kB. According to PI3K-Akt-mTOR pathway in KEGG pathway analysis [33], rac1/cdc42 shown to be connected with TLR4, which is a common receptor for HMGB1 [34]. Then we speculate that HMGB1 may induce the expression of RAC1 and CDC42 followed by binding to TLR4, and activate PI3K/AKT signaling pathway, which plays an important role in cancer cell growth and malignant transformation. The pathway-related networks provide us with a number of potential genes or pathways that may relate to proliferation and metastasis functions of HMGB1 in cervical cancer; hence, the call for further investigations.

The limitation in our study may be that molecular functions of HMGB1 towards cervical cancer were analyzed mostly by bioinformatics analysis, the results would be diverse when divided by different criteria for grouping of HMGB1 expression and analyzed by different databases. Therefore, the specific mechanism of the interaction among HMGB1, RAC1, and CDC42 should be further investigated with some following studies.

In summary, we suggest that HMGB1 expression was independent predictors of shorter OS and DFS in patients with cervical cancer. RAC1 and CDC42 may involve in the progression of cervical cancer migration as downstream of HMGB1. Results of the present study may help to further understand the effects of HMGB1 in cervical cancer during its progression and how it may lead to poor survivorship and reveal potential targets for diagnostic and therapeutic manipulation.

## Abbreviations

CIN: Cervical Intraepithelial Neoplasia
DAVID: Database for Annotation, Visualization and Integrated Discovery
DEG: differentially expressed gene
DFS: disease free survival
FIGO: International Federation of Gynecology and Obstetrics
GAPDH: glyceraldehyde-3-phosphate dehydrogenase
GEO: Gene Expression Omnibus
GO: Gene Ontology
HMGB1: high-mobility group box 1 protein
HPV: human papillomavirus
IHC: immunohistochemistry
JNK: c-Jun N-terminal Kinase
KEGG: Kyoto Encyclopedia of Genes and Genomes
MAPK: mitogen-activated protein kinase
NC: negative control
OS: overall survival
PPI: protein–protein interaction
qRT-PCR: quantitative real-time PCR
STRING: Search Tool for the Retrieval if Interacting Gene
