# Correction: The effect of HMGB1 on the clinicopathological and prognostic features of cervical cancer

**DOI:** 10.1042/BSR-20181016_COR

**Published:** 2020-06-19

**Keywords:** cervical cancer, HMGB1, prognosis, bioinformatics

The authors of the original article above article “The effect of HMGB1 on the clinicopathological and prognostic features of cervical cancer” (*Bioscience Reports* (2019) **39**, https://doi.org/10.1042/BSR20181016) would like to correct Figure 2A, as the survivor curves of Overall survival and Disease-free survivor were repeated due to mis-operation in visualisation. The authors had mistakenly reused the image of OS for DFS. The authors declare that these corrections do not change the results or conclusions of this paper, and express their sincere apologies for any inconvenience that this error has caused to the readers. The corrected version of Figure 2 is presented here.

**Figure 2:**
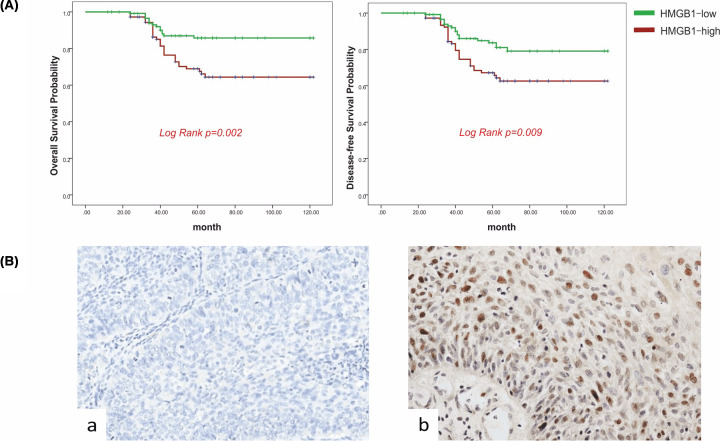
Clinical-pathological analysis (**A**) Survival curves using the Kaplan–Meier method by log-rank test for HMGB1 expression. OS rate was analyzed in 239 cervical cancer patients in relation to HMGB1 expression. Log-rank test P-value: 0.002. And disease-free time rate were analyzed in 239 cervical cancer patients in relation to HMGB1 expression. Log-rank test P-value: 0.009. (**B**) Representative immunohistochemistry samples of cervical cancer tissues demonstrating HMGB1 expression. Normal cervix tissue showed the negative HMGB1 staining reactivity. And cervical cancer tissue showed the positive HMGB1 staining reactivity. Magnification, ×400.

